# Metamorphosis of a Butterfly-Associated Bacterial Community

**DOI:** 10.1371/journal.pone.0086995

**Published:** 2014-01-23

**Authors:** Tobin J. Hammer, W. Owen McMillan, Noah Fierer

**Affiliations:** 1 Department of Ecology and Evolutionary Biology and Cooperative Institute for Research in Environmental Sciences, University of Colorado at Boulder, Boulder, Colorado, United States of America; 2 Smithsonian Tropical Research Institute, Panama City, Republic of Panama; Ghent University, Belgium

## Abstract

Butterflies are charismatic insects that have long been a focus of biological research. They are also habitats for microorganisms, yet these microbial symbionts are little-studied, despite their likely importance to butterfly ecology and evolution. In particular, the diversity and composition of the microbial communities inhabiting adult butterflies remain uncharacterized, and it is unknown how the larval (caterpillar) and adult microbiota compare. To address these knowledge gaps, we used Illumina sequencing of 16S rRNA genes from internal bacterial communities associated with multiple life stages of the neotropical butterfly *Heliconius erato*. We found that the leaf-chewing larvae and nectar- and pollen-feeding adults of *H. erato* contain markedly distinct bacterial communities, a pattern presumably rooted in their distinct diets. Larvae and adult butterflies host relatively small and similar numbers of bacterial phylotypes, but few are common to both stages. The larval microbiota clearly simplifies and reorganizes during metamorphosis; thus, structural changes in a butterfly's bacterial community parallel those in its own morphology. We furthermore identify specific bacterial taxa that may mediate larval and adult feeding biology in *Heliconius* and other butterflies. Although male and female *Heliconius* adults differ in reproductive physiology and degree of pollen feeding, bacterial communities associated with *H. erato* are not sexually dimorphic. Lastly, we show that captive and wild individuals host different microbiota, a finding that may have important implications for the relevance of experimental studies using captive butterflies.

## Introduction

Butterflies are important herbivores and pollinators and are used as model systems in a variety of ecological and evolutionary fields [1]. Like all animals, butterflies also host internal communities of microorganisms, yet their associations with these symbionts remain poorly understood. This knowledge gap persists despite a large and rapidly growing body of work on other insect groups demonstrating that microbes can have important effects on host nutrition, digestion, detoxification, and defense from predators, parasites, and pathogens [2]–[5]. Studies of butterfly-associated microorganisms therefore have the potential to advance our understanding of the biology of butterflies and their ecological and evolutionary interactions with plants and natural enemies.

Unfortunately, even basic information on butterfly microbial symbionts is lacking, making it difficult to identify the potential impacts that these microbes may have on butterfly ecology and evolution. While various bacteria have been isolated from the adult butterfly intestinal tract [6], [7], and the presence of *Wolbachia* and *Spiroplasma* has been reported in the adults of some species [8]–[10], there are no community-level descriptions of the dominant microbial taxa present. Kingsley [11] cultured multiple bacterial populations from the gut of newly emerged adult monarch butterflies, but such cultivation-based surveys are well known to misrepresent the community structure *in situ* [12]. To our knowledge, there have been no previous culture-independent studies of microbial communities associated with adult butterflies.

Additionally, while the larval gut microbiota of a handful of butterfly species have been described [13], [14], it is not known how microbial communities associated with larvae compare with those in the adult stage, nor how they may change during metamorphosis. In fact, this question has not been addressed in any lepidopteran since the advent of molecular tools for characterizing microbial diversity. Kingsley's survey of monarch gut bacteria [11] included multiple developmental stages, but owing to a dependence on culturing and physiology-based taxonomic assignments, it is uncertain whether those findings are generalizable. We do know from work on other holometabolous insect groups that larvae may have few or no microbial symbionts [15], [16], different microbiota [17]–[19], or similar microbiota as adults [20], [21]. We expected that butterfly larvae and adults would host distinct bacterial communities owing to the radical switch in diet from the larval to the adult stage of butterflies, as well as the changes in internal morphology and physicochemical conditions that accompany metamorphosis. Diet is a major factor structuring microbiota across animal taxa [22], [23], and diet shifts may also underlie patterns of microbial variation across developmental stages of a single host. For example, nutritional or chemical differences between the diets of larvae and adults may differentially select for microbial taxa best able to grow at each stage. Conversely, those particular microbial taxa may aid the host in utilizing life-stage-specific resources by providing functions related to digestion, detoxification, and/or nutrient supplementation.

Perhaps the most striking contrast in feeding biology between butterfly larvae and adults is in the neotropical genus *Heliconius*. *Heliconius* larvae consume leaves and stems of cyanogenic glycoside-rich passion-flower vines [24], while adults visit flowers to feed on pollen as well as nectar. Among butterflies, pollen feeding is an evolutionary innovation unique to *Heliconius*, and has led to major changes in reproductive biology and life history traits [25]. We therefore focused on *Heliconius* to test for a possible differentiation in microbial community structure between the larval and adult stages. Additionally, *Heliconius* butterflies represent an ideal model system for microbial symbiosis research as they are collectable in the wild and experimentally tractable, and as a wide array of relevant ecological, evolutionary, and genomic information is available [27], [28]. In contrast, almost nothing is known about their microbiota, besides the sporadic presence of *Wolbachia* [28], [29]. Given their distinctive larval and adult diets, *Heliconius* butterflies also provide an opportunity to test whether associations with microbial symbionts have been important in the evolution of host traits related to herbivory and pollen feeding.

In addition to investigating how *Heliconius*-associated microbial communities change across different life stages, we also wanted to determine how wild and captive *Heliconius* butterflies may differ with respect to their microbiota. Many experimental studies of *Heliconius* (and other butterflies) have used lab- or insectary-reared subjects. Evidence from moth larvae [30], [31] and other insects [32], [33] suggests that symbiont community structure can change when hosts are brought from the wild into captivity, an effect possibly mediated by artificial diets or selection history. Testing whether captive and wild butterflies are different in terms of their microbiota is important not only for future microbial investigations, but also for other types of studies on captive butterflies where the phenomena under question may be influenced by microbial symbionts (including, but not limited to, host plant use and defense against parasites or parasitoids).

We used a high-throughput DNA sequencing-based approach to characterize internal bacterial communities associated with the butterfly *Heliconius erato*, thus providing a foundation for future studies of microbial symbiosis in *Heliconius* and other butterflies. To test the hypothesis that the microbiota varies across the butterfly life cycle, we compared bacterial community structure in replicate larvae, pupae, newly emerged adults, and mature adults of *H. erato*. We also assessed variation in bacterial community diversity and composition between wild adults sampled from the field, wild adults maintained in an insectary, and the reared adult offspring of the latter to determine whether captive butterflies harbor bacterial communities representative of their wild counterparts.

## Methods

### Insect collection and rearing

In April and May 2012, adult *Heliconius erato* butterflies were collected from a wild population as they visited flowers in Parque Nacional Soberanía, Panama (9°7′20″N, 79°42′54″W), for which permission was provided by the Panamanian Environmental Authority (ANAM) under permit #SE/A-92-11. Voucher specimens have been deposited at the Fairchild Invertebrate Museum of the University of Panama. Thirteen individuals (nine males and four females) were stored at −20°C directly after field collection. All samples described below were preserved in the same manner.

We relocated nine additional wild-caught females to a nearby insectary, where they were housed under semi-natural conditions in separate mesh cages. They were supplied with flowers frequently visited by wild *H. erato* in this area (*Psychotria elata*, *Lantana camara*), and with an autoclaved sucrose and honeybee pollen solution. Potted *Passiflora biflora*, the main host plant of the specialist *H. erato* [34], were placed in the cages to elicit oviposition. Eggs were removed and placed individually in plastic cups. The parental females were sampled after a sufficient number of eggs were obtained, corresponding to appx. 2–4 weeks in captivity. Given that females of *H. erato* only very rarely mate more than once in the wild [35], it is likely that the individuals in each brood are full siblings.

We reared larvae on plant material collected from potted *P. biflora* grown in an open-air greenhouse near the forest. One larva per brood was sampled two days into the fifth stadium, while it was actively feeding, as was the frass it had produced that day. Pupae were sampled midway through the pupal stage. Newly emerged adults were sampled immediately after they had excreted meconium. The rest of the adults were kept under identical conditions as described above for wild-caught parental females. One male and one female per brood were sampled four days after eclosion, by which point both sexes of this species have reached sexual maturity.

### Sample processing

We used whole, surface-sterilized insects to describe the dominant bacterial taxa associated with the internal portion of the body. Insects were rinsed in sterile molecular-grade water (Sigma-Aldrich), soaked in 70% ethanol for 30 s followed by 10% bleach for 30 s, and rinsed again in sterile water. For adults, wings were clipped where they met the thorax prior to sterilizing the body. After surface sterilization the samples were ground under liquid N_2_ with single-use, sterile mortar and pestles (Fisher Scientific). Frass samples were not surface sterilized.

### DNA sequencing and data processing

Bacterial communities were characterized using barcoded Illumina sequencing of 16S rRNA genes. Total DNA was extracted from homogenized material using the MoBio PowerSoil kit as described previously [36]. We used the primer pair 515F/806R to amplify the V4 region of the 16S rRNA gene, and PCR conditions followed those described previously [37]. Amplicons were sequenced on the Illumina MiSeq platform, resulting in an average of 1779 150-bp reads per sample after filtering with default parameters for sequence length and minimum quality score in QIIME v. 1.6.0 [38]. Sequences were clustered into operational taxonomic units (hereafter, “phylotypes”) at the 97% similarity level by reference-based picking with the QIIME implementation of UCLUST [39] against the October 2012 release of the Greengenes database [40] with remaining sequences clustered *de novo*. The Ribosomal Database Project (RDP) classifier [41] set at a minimum confidence level of 0.5 was used to assign taxonomy to the phylotypes. The centroid (seed sequence) used by UCLUST was chosen as the representative sequence for each phylotype. With representative sequences from the 10 most abundant phylotypes across all *H. erato* samples, we used SeqMatch to find the best high-quality matches ≥1200 bp in the curated RDP 16S database [42].

Because this primer set can amplify non-bacterial rRNA gene sequences, phylotypes identified by the RDP classifier as chloroplast or mitochondrial 16S rRNA (which represented 24% of the sequences on average) were removed prior to downstream analyses. In order to standardize sequencing effort, all samples were rarefied by randomly selecting 500 sequences per sample. As the samples from which we obtained fewer than 500 bacterial sequences were excluded from further analysis, there are fewer replicates for pupae than were initially collected. This sequencing depth has been shown to be sufficient for detecting biological patterns in insect-associated bacterial communities [43] and other community types [44]. Amplicon sequences and associated metadata from this study are publicly available in the EMBL-EBI database (http://www.ebi.ac.uk/) under accession number ERP003400.

### Statistical analyses

We used nonparametric Kruskal-Wallis tests in R v. 3.0.0 [45] to determine whether there were significant differences in community richness or the relative abundances of individual bacterial taxa (families or phylotypes) with a Bonferroni correction applied to account for multiple comparisons. The family-level tests were conducted only on dominant families, defined as those contributing at least a median 2% of the sequences within any of the factor levels. To compare community composition between sample types, we used vegan [46] to compute a Bray-Curtis dissimilarity matrix after Hellinger transformation of the phylotype count data. Subsequent multivariate analyses were conducted in PRIMER [47]. Variation among samples in their bacterial taxonomic composition was visualized using constrained principal coordinates analyses [48]. We used Mantel tests to determine whether patterns of compositional dissimilarities among larvae were correlated with dissimilarities among their frass. Permutational multivariate ANOVA tests [49] were used to assess differences in bacterial community composition associated with several sample categories, with tests of life stage or frass versus larvae run using sample type as a fixed effect. Variation in the dissimilarity matrix linked to the level of relatedness among captive adults was tested using family as a random effect. Lastly, for all adult butterflies, a two-factor design was used to test the effects of captivity/rearing status and sex (both fixed).

## Results

### Bacterial community dynamics across the life cycle

**Figure 1:**
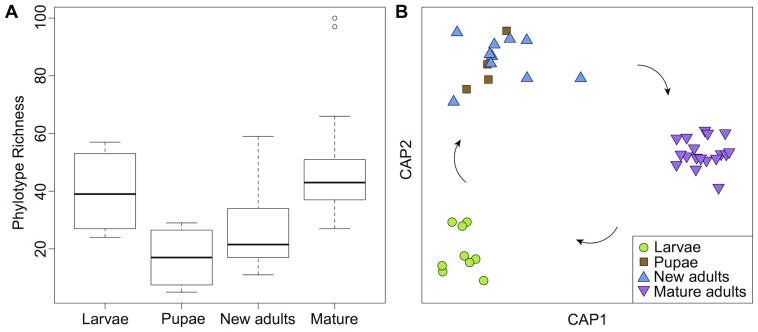
Bacterial community dynamics across *H. erato* larvae, pupae, newly emerged adults, and mature adults. A. Boxplot of community phylotype richness. B. Constrained principal coordinates analysis showing variation in community composition over the life cycle. CAP1 and CAP2 are the canonical axes in principal coordinate space that best discriminate among life stages. Arrows indicate significant pairwise differences in composition.

Bacterial phylotype richness varied among life stages (Fig. 1A, *P*<0.01). Median richness was similar between larvae and mature adults with 39 and 43 phylotypes per individual, respectively. In contrast, pupae and newly emerged adults were associated with roughly half as many phylotypes (median 17 and 22 phylotypes, respectively). Nearly identical patterns were observed when diversity was measured using the Shannon index, which takes relative abundances into account (Fig. S1, *P*<0.01). A comparison restricted to only the numerically dominant phylotypes–those contributing at least 5 sequences per sample (1%)–produced a similar pattern: median richness of dominant phylotypes was 12 in both larvae and mature adults, and 4 and 5.5 in pupae and newly emerged adults, respectively.

Bacterial community composition also varied across life stages (Fig. 1B, *P* = 0.001). In agreement with the pattern shown in the constrained ordination (Fig. 1B), all pairwise comparisons were significant at *P*<0.05 except that between pupae and newly emerged adults. On average, only 13% of the phylotypes present in either the larva or mature adults of each replicate brood were present in both stages.

Communities from frass samples and the individual larvae that produced them were not significantly different in composition (Fig. S2, *P* = 0.16). Additionally, variation in community composition among larvae was reflected in their frass (Fig. S2, *P*<0.05, Mantel rho  = 0.47).

The four life stages of *H. erato* analyzed here were dominated by six bacterial families: the Acetobacteraceae (Alphaproteobacteria), Moraxellaceae and Enterobacteriaceae (Gammaproteobacteria), Enterococcaceae and Streptococcaceae (Firmicutes), and an unclassified family in the Bacteroidetes phylum (Fig. S3). Although family-level bacterial community composition varied substantially between individuals of the same life stage in some cases, all of these families excluding the Enterococcaceae and Enterobacteriaceae shifted significantly in relative abundance across the life cycle (Fig. S3, Bonferroni-corrected *P*<0.05).

The 10 most abundant phylotypes present across all *H. erato* samples are listed in Table 1. The split between larval and adult communities appears to be driven by the higher relative abundance of *Acinetobacter* in the larvae and of *Asaia*, *Lactococcus*, and an unclassified Bacteroidetes phylotype in the mature adults. Most of these phylotypes matched at 98–100% identity to named isolates in the RDP database. Two phylotypes had highest similarity to sequences obtained from uncultured bacteria in ground beetle and honeybee digestive tracts.

**Table 1:** **Dominant bacterial phylotypes in** ***H. erato*** **.**

Taxonomic classification	% Total	Larvae (9)	Pupae (4)	New adults (10)	Mature adults (18)	Parental adults (9)	Wild adults (13)	Best-scoring RDP match	% ID
Firmicutes (Enterococcaceae)	16.04	16.96	67.70	31.40	9.17	3.78	5.72	*Enterococcus*	100
γ-Proteobacteria‡ (Enterobacteriaceae)	15.17	18.07	2.45	37.92	19.96	2.22	1.92	*Enterobacter*	100
γ-Proteobacteria (Enterobacteriaceae)	6.86	9.18	0.10	1.00	7.34	8.51	10.02	*Enterobacter*	100
Bacteroidetes** (Unclassified)	5.36	0.02	0.00	1.12	10.70	8.78	4.22	Unclassified (honeybee gut)	94.7
γ-Proteobacteria† (Orbaceae)	4.83	0.02	0.00	1.68	2.07	4.04	16.46	*Orbus*	100
Firmicutes** (Streptococcaceae)	4.50	0.69	0.00	3.56	7.93	5.73	3.62	*Lactococcus*	100
α-Proteobacteria‡ (Acetobacteraceae)	4.45	0.07	0.05	0.58	0.64	17.02	8.38	*Commensalibacter*	98
γ-Proteobacteria** (Moraxellaceae)	3.91	22.33	8.15	0.94	0.06	0.27	0.02	*Acinetobacter*	100
α-Proteobacteria* (Acetobacteraceae)	2.98	0.00	0.00	0.90	5.66	3.16	3.72	*Asaia*	100
γ-Proteobacteria (Pseudomonadaceae)	2.41	0.00	0.00	0.04	0.00	2.49	9.92	Unclassified (ground beetle gut)	99.3

The 10 most abundant bacterial phylotypes (by percent of total sequences) across all *H. erato* samples, with phylum (or class for Proteobacteria) and family-level classification. The number of specimens sequenced per sample type is indicated in parentheses. Mean percent relative abundances are shown for each life stage and adult group. The genus-level taxonomic identification of the best match for each phylotype, using the RDP SeqMatch tool, is shown along with its percent sequence identity. If the best match is unclassified, the habitat from which it was sequenced is given.

Asterisks indicate significantly different relative abundances across life stages (Bonferroni-corrected *P*<*0.01, **0.001). The † symbols indicate significantly different relative abundances across adult groups (Bonferroni-corrected *P*<†0.01, ‡0.001).

### Factors structuring adult-associated microbiota

**Figure 2:**
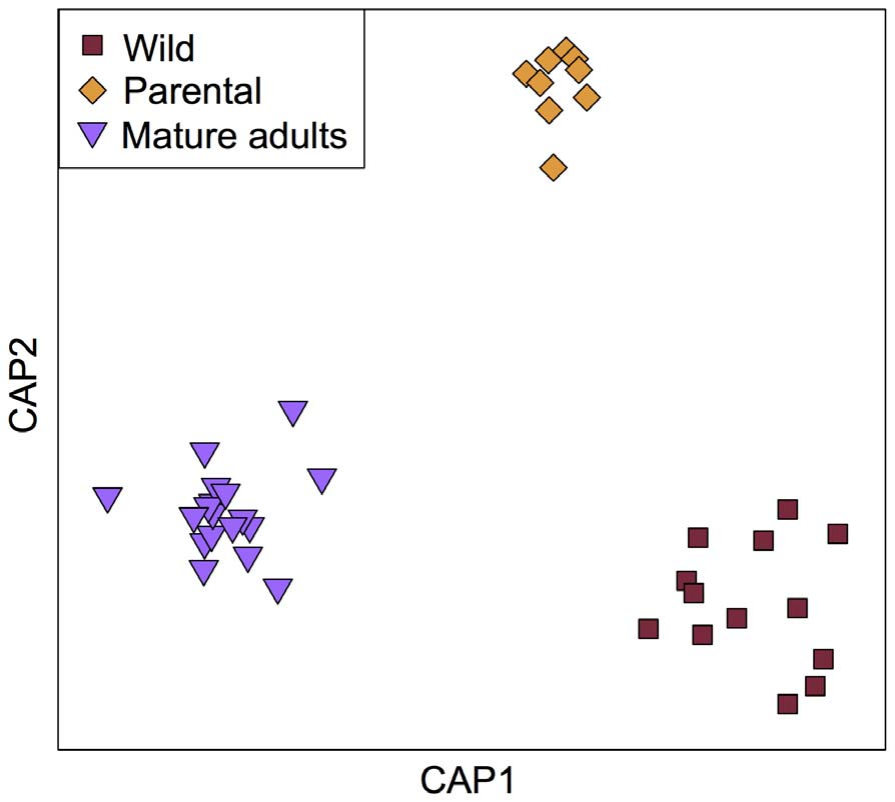
Bacterial community variation associated with captivity and rearing. Constrained principal coordinates analysis showing differences in community composition between adults sampled directly from the wild, wild-caught females kept in an insectary (“Parental”), and their reared adult offspring (“Mature adults”). CAP1 and CAP2 are the canonical axes in principal coordinate space that best discriminate among adult groups.

Bacterial phylotype richness did not differ between wild, captive wild-caught (parental), and reared mature adult butterflies (*P* = 0.24), although each group hosted bacterial communities distinct in composition (Fig. 2, *P* = 0.001; all pairwise comparisons significant at *P*<0.05). Despite compositional differences, all adults clustered together to the exclusion of reared larvae (Fig. S4).

Four of the six dominant adult-associated bacterial families differed in relative abundance between the three groups we analyzed (Fig. S5, Bonferroni-corrected *P*<0.05). Specifically, an increase in Streptococcaceae and reduction in an unclassified Gammaproteobacterial family were associated with captivity, whereas an increase in Enterobacteriaceae and reduction in Acetobacteraceae were associated with rearing. Among all adult butterflies, sex did not have an effect on community composition (*P* = 0.80), and there was no interaction between sex and captivity/rearing status (*P* = 0.33). Among the butterfly individuals with known relatedness (i.e., captive females and their mature adult offspring), variation between families was not greater than variation within families (*P* = 0.78).

## Discussion

### Overall structure of the Heliconius erato microbiota

*Heliconius erato* larvae and adult butterflies host relatively simple bacterial communities, in agreement with previous reports of low diversity in other lepidopterans [14], [50], [51] and other insect orders [21], [43] relative to vertebrate-associated and free-living microbiota. The uneven structure of these communities is illustrated by the observation that the 10 most abundant phylotypes contributed more than 65% of the sequences from all *H. erato* samples. The majority of these dominant phylotypes were highly similar to sequences from genera known to colonize the gut of lepidopterans and other insects. The phylotype with the highest abundance across all *H. erato* samples matched most closely to isolates in the genus *Enterococcus*. Enterococci are commonly present in the intestinal tract of lepidopteran larvae [13], [50], [52] and other insects [53], but are also found free-living in a variety of environmental habitats [54]. Evidence from other lepidopterans that enterococci in the larval gut can persist through metamorphosis [55] is supported by our finding that *Enterococcus* is prevalent in all stages of *H. erato*.

A phylotype matching with 100% sequence identity to an *Orbus* clone in the Orbaceae was also abundant. Although the natural history of this family is not well known, one member has been isolated from a butterfly gut [7], and two others are associated with the gut of honeybees [56], [57]. Another phylotype classified as *Acinetobacter* was variably present across life stages, but at highest relative abundance in the larvae. *Acinetobacter* sequences have been reported from the larval midgut of a number of insect species including cabbage white butterflies [14] and saturniid moths [58], although their possible role in host herbivory is not well understood.

Phylotypes belonging to the bacterial family Acetobacteraceae were overrepresented in mature adults relative to earlier stages (Fig. S3). Bacteria in this family are commonly associated with the intestinal tract of insects with sugar-rich diets, such as adult mosquitoes, bees, fruit flies, and sugarcane mealybugs [59]. We discovered two dominant Acetobacteraceae phylotypes in *H. erato*, one of which matches to *Asaia sp.*, which in other insects can form biofilms on the midgut epithelium and colonize egg surfaces and reproductive structures [60]. As members of the *Drosophila* gut flora, acetic acid bacteria have been shown to prevent colonization by pathogens [61], affect development and insulin signaling [62], and influence dietary carbohydrate utilization [63]. Such bacteria are likely to be broadly associated with nectar- and fruit-feeding adult butterflies, in which they may have similar functions, and their role in the biology of *Heliconius* clearly warrants further investigation.

Another dominant phylotype in the adult stage, a member of the Bacteroidetes phylum, appears to be only distantly related to taxa reported from insects or other habitats. Interestingly, its closest match was to a clone from honeybee intestines [64]. We do not know if this phylotype is uniquely associated with *Heliconius*, but given that a similar bacterium has been found in honeybees, which also feed on pollen, it is possible that this taxon is involved in *Heliconius* pollen feeding. For example, certain honeybee gut bacteria can produce enzymes that degrade pectin, a major structural component of pollen walls [65]. In *Heliconius*, which digest pollen grains attached to the proboscis using exuded saliva [66], symbionts with similar functions could reside in the salivary gland.

Because we sampled the entire internal portion of the insect, the exact location of these taxa within the host is unknown. Bacteria could reside in other structures besides the gut, such as reproductive organs and the salivary gland. However, the observation that frass samples were not different in composition from the whole larvae that produced them indicates that, for the larval stage at least, we have primarily sequenced gut bacteria. Likewise, previous studies have found that communities from whole homogenized insects can closely resemble those sampled from the gut alone [67], [68].

### Effects of captivity and rearing on adult butterfly microbiota

Studies of microbial symbionts in Lepidoptera and other insects commonly use hosts reared in the laboratory where they are often maintained for multiple generations on artificial diets. We found that *H. erato* butterflies sampled directly from the wild were different in bacterial community composition from individuals from the same population housed in an insectary for 2–4 weeks. Although the reasons for this microbial community shift remain unknown, altered adult diet–specifically, access to artificial sucrose/pollen solution, and the absence of certain flowers normally visited by *H. erato* in the wild–could underlie this difference, as could altered exposure to microbial inocula from their environment.

Reared four-day-old adult offspring were also different in composition from their wild-caught mothers, despite being maintained under identical conditions in the insectary. Although the average age of the wild-caught group is unknown, a difference in adult age could be partly responsible for these differences. As the wild-caught mothers spent all of the larval stage and some period of the adult stage in the wild prior to capture, there could be additional effects of diet and exposure to microbial inocula in both stages.

Generally, these results support previous findings of captive-wild differences in insect-associated microbial communities [30]–[33] and they suggest that caution should be taken when inferring evolutionary history or ecological function from microbiota associated with captive insects without an explicit comparison to wild populations. Altered bacterial community composition in captive individuals may also affect host nutrition, detoxification, and defense from natural enemies, as these traits can be mediated by microbial symbionts. The use of captive experimental subjects may consequently render studies of these phenomena less relevant to natural conditions. Although not tested here, these changes in the microbiota could partly account for the observations that reared *Heliconius* butterflies exhibit lower success in courtship and pollen collection compared with wild-caught individuals [26].

### Community dynamics across metamorphosis

Bacterial diversity dropped by approximately 50% from the larval to the pupal stage, remained low in the newly emerged adults, and redoubled in the mature adults after feeding. Likewise, bacterial communities changed in composition from the larval to the pupal stage, remained similar in the newly emerged adults, and changed again in the mature adults. Thus, butterfly-associated bacterial communities appear to both simplify and reorganize over metamorphosis, a pattern that can be explained by multiple possible mechanisms. The reduction in richness during metamorphosis could be due to larval voiding of the gut prior to pupation [69] and/or secretion of antibacterial proteins into the pupal gut lumen [70], both of which could selectively eliminate or reduce the abundance of gut-associated bacteria. Degeneration of the larval gut and its contents, in tandem with the development of a morphologically distinct adult gut [71]–[73] and new structures such as the adult salivary gland and reproductive organs, could also facilitate the successional patterns observed here. After adult emergence, feeding by the host might stimulate the growth of bacteria persisting through the pupal stage, or add new taxa sourced from the diet, restoring community richness–though not composition–to pre-metamorphosis levels.

Differences in diet presumably drive the remarkable difference in bacterial community composition between *H. erato* larvae and adults (Figs. 1B and S4). Diet could directly impact life-stage-specific microbiota as an inoculum, as a resource supporting the differential growth of resident bacteria, and as a source of chemical compounds with selective antimicrobial activity. Diet may also directly affect the environmental conditions within the host–for example, by inducing gut pH changes [74], [75]. Additionally, diet could indirectly impact the microbiota through the morphological and biochemical adaptations hosts have evolved to utilize different resources in different life stages (here, foliage versus nectar and pollen).

### Impact of holometaboly on insect microbiota

The spectacular success of the Holometabola, of which the Lepidoptera are one of the most diverse groups [76], has been attributed to the differentiation in form and function between larvae and adults [77]. This divergence enables specialization on different diets in the larval and adult stages and reduces competition between immature and mature conspecifics for resources [78], [79]. We propose that the evolutionary innovation of holometaboly also created distinct niches for colonization by distinct microbial symbionts. Over the holometabolous host life cycle, variation in diet and internal physicochemical conditions could support communities functionally specialized for a particular life stage. It remains to be determined whether holometabolous species–especially those whose adults feed, and on diets distinct from the larvae–are thus associated with more diverse microbial symbiont communities than other insects.

## Conclusions

We have identified a relatively simple bacterial community associated with *H. erato* that differs in composition between larvae and adults. This difference in taxonomic membership may reflect divergent functional roles in life-stage-specific resource use. These results will be valuable in designing genomic studies and experimental manipulations to test how *Heliconius*-associated bacteria may be involved in their host's distinctive feeding biology. Additionally, the overall compositional similarity between frass and whole larvae, as well as the finding that community differences among larvae are maintained in their frass, indicate that frass could be used in the future as a way to sample the larval gut microbiota nondestructively. As with temporal surveys of the human gut [80], this would allow an analysis of gut communities from the same individual over larval development and into the adult stage.

Furthermore, we found that both captivity and rearing are associated with a compositional change in the microbiota from wild *H. erato* individuals of the same population. This change could be partly responsible for observed differences in performance between wild-caught and captive butterflies, and has implications not only for future studies of butterfly symbionts, but also for other kinds of studies on captive butterflies where microbial differences may influence experimental results.

We have demonstrated that the internal bacterial community of *H. erato* simplifies and reorganizes across host development. Presumably, different life stages represent habitats that selectively favor the growth of certain bacterial taxa. This ability of the microbiota to undergo a structural “metamorphosis,” in tandem with its host, might entail an overall greater diversity in microbial community form and function within a given holometabolous species relative to other insect groups.

## Supporting Information

Figure S1 **Changes in bacterial community diversity across life stages.** Boxplot of Shannon Diversity Index values from *H. erato* larvae, pupae, newly emerged adults, and mature adults, standardized at 500 sequences per sample.
(TIFF)

Figure S2 **Clustering patterns of larval and frass communities.** Principal coordinates analysis of bacterial communities in whole larvae and their frass, colored by individual, showing clustering by individual rather than sample type.
(TIFF)

Figure S3 **Dynamics of bacterial families across life stages.** Relative abundances of the six dominant bacterial families among *H. erato* life stages, defined as those with a median abundance over 2% within any life stage. Points represent individual samples and are laterally jittered to display within-stage variability more clearly. Bars show median relative abundances. Asterisks indicate bacterial families whose relative abundances differed significantly across life stages.
(TIFF)

Figure S4 **Clustering pattern of bacterial communities from multiple adult groups and reared larvae.** Constrained principal coordinates analysis of bacterial community composition in *H. erato* larvae and all adult groups. CAP1 and CAP2 are the axes in principal coordinate space that best discriminate among sample types.
(TIFF)

Figure S5 **Dynamics of bacterial families across wild, captive, and reared adults.** Relative abundances of the six dominant bacterial families among *H. erato* adult groups, defined as those with a median abundance over 2% within any group. Points represent individual samples and are laterally jittered to display within-group variability more clearly. Bars show median relative abundances. Asterisks indicate bacterial families whose relative abundances differed significantly across groups.
(TIFF)
