# Supplementary figures and images for: Metamorphosis of a Butterfly-Associated Bacterial Community
Source: PLoS One. 2014 Jan 23;9(1):e86995. doi: 10.1371/journal.pone.0086995 (PMC3900687; doi:10.1371/journal.pone.0086995)

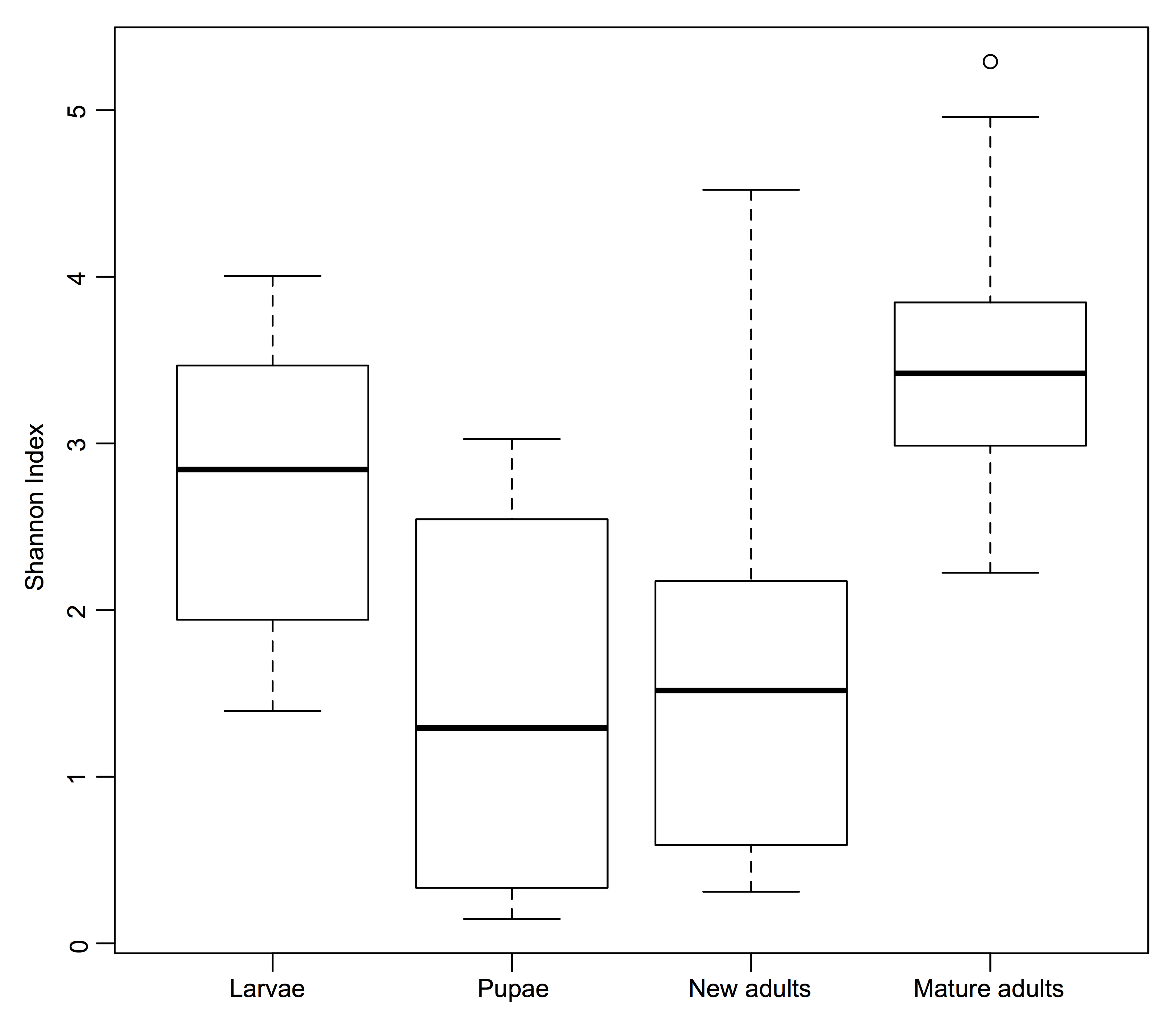

Supplement: Figure S1 — Changes in bacterial community diversity across life stages. Boxplot of Shannon Diversity Index values from H. erato larvae, pupae, newly emerged adults, and mature adults, standardized at 500 sequences per sample. (TIFF) [file pone.0086995.s001.tiff]

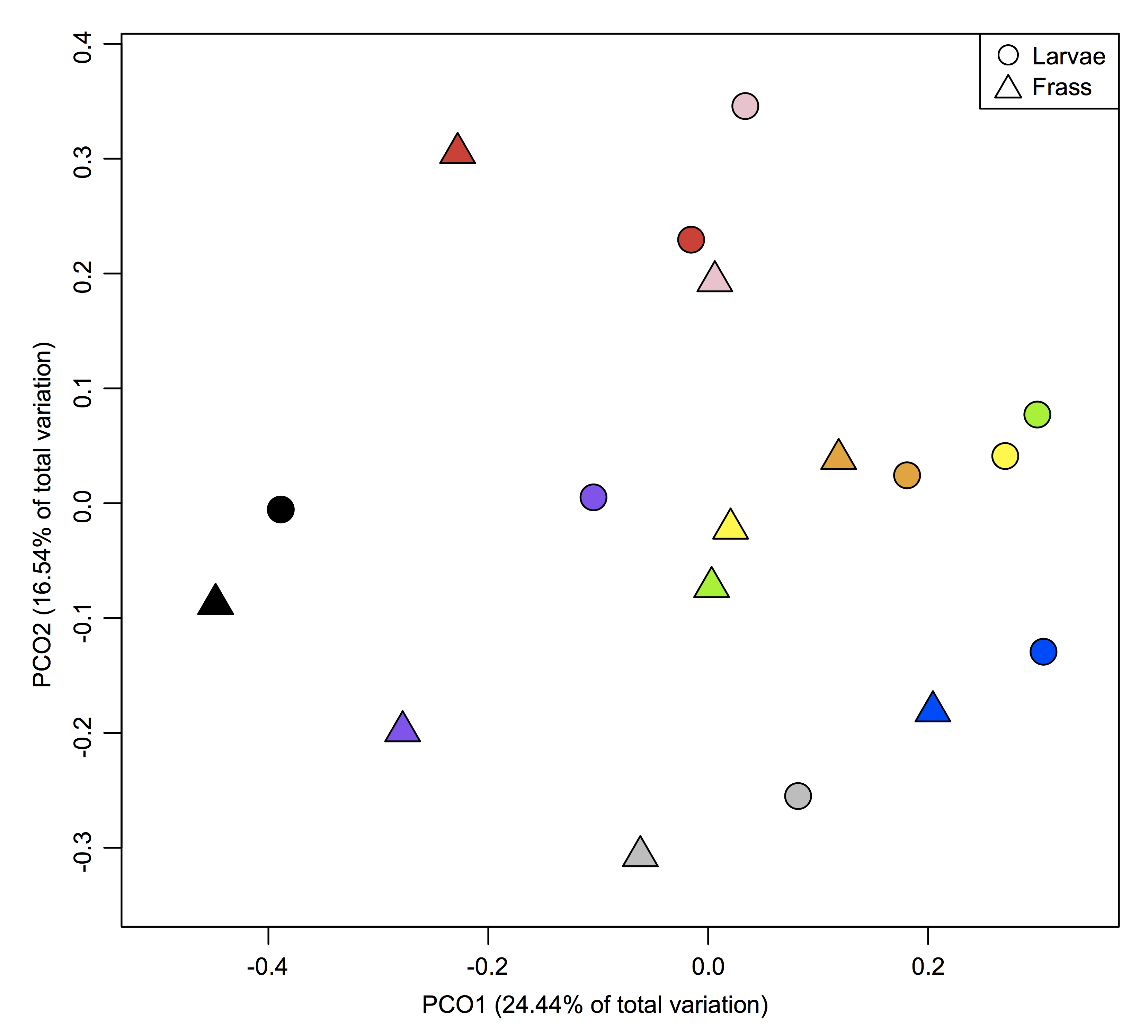

Supplement: Figure S2 — Clustering patterns of larval and frass communities. Principal coordinates analysis of bacterial communities in whole larvae and their frass, colored by individual, showing clustering by individual rather than sample type. (TIFF) [file pone.0086995.s002.tiff]

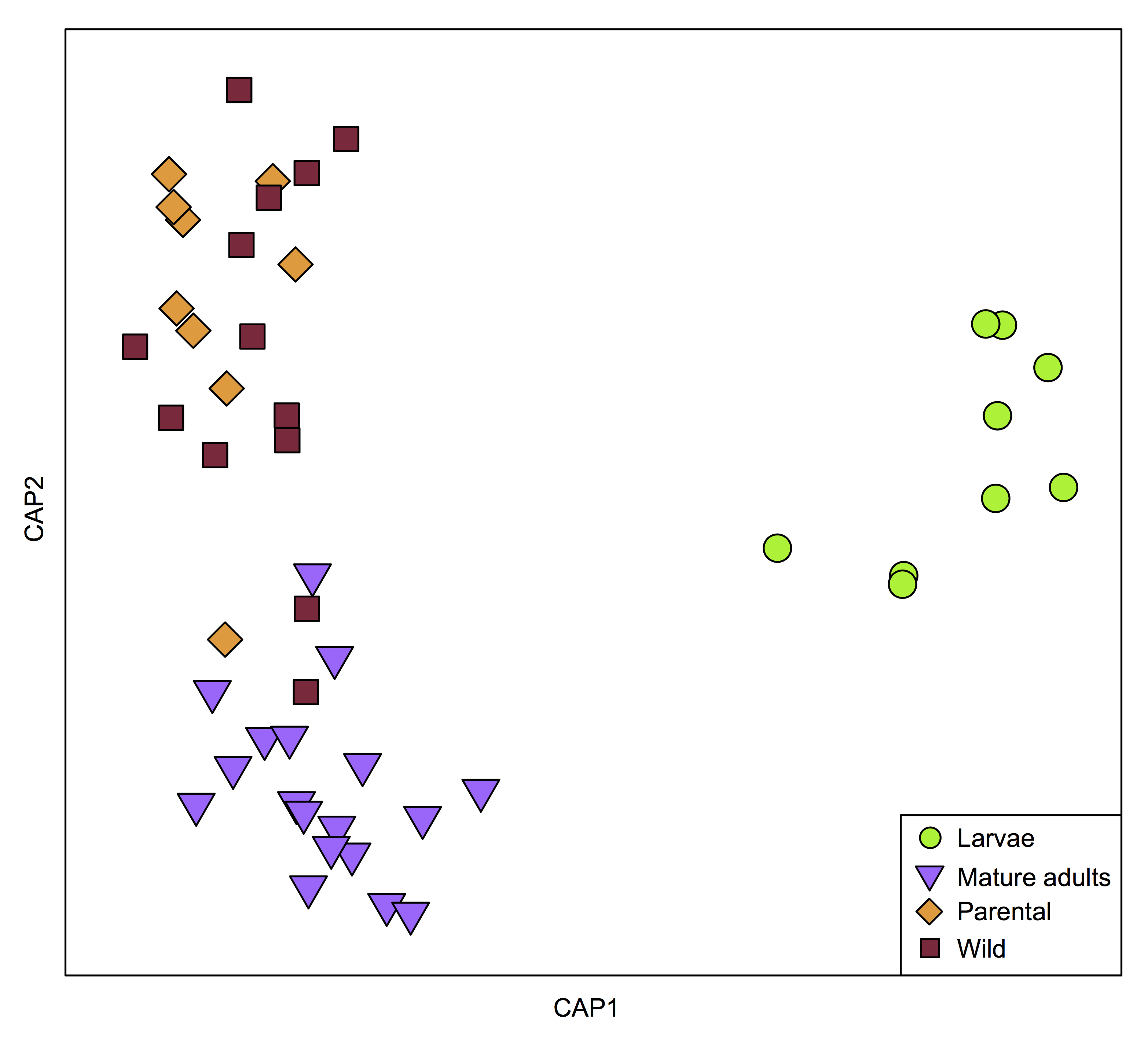

Supplement: Figure S4 — Clustering pattern of bacterial communities from multiple adult groups and reared larvae. Constrained principal coordinates analysis of bacterial community composition in H. erato larvae and all adult groups. CAP1 and CAP2 are the axes in principal coordinate space that best discriminate among sample types. (TIFF) [file pone.0086995.s004.tiff]
